# Positionspapier der Initiative Chronische Wunde (ICW) e. V. zur Nomenklatur des Débridements chronischer Wunden

**DOI:** 10.1007/s00105-022-04944-3

**Published:** 2022-01-24

**Authors:** Joachim Dissemond, Anke Bültemann, Veronika Gerber, Martin Motzkus, Karl Christian Münter, Cornelia Erfurt-Berge

**Affiliations:** 1 Klinik und Poliklinik für Dermatologie, Venerologie und Allergologie, Universitätsklinikum Essen, Hufelandstr. 55, 45122 Essen, Deutschland; 2 Klinik für Gefäß- und Viszeralchirurgie, Asklepios Klinikum Harburg, Hamburg, Deutschland; 3 Initiative Chronische Wunde (ICW) e. V., Quedlinburg, Deutschland; 4 Zentrales Wundmanagement, Evangelisches Krankenhaus Mülheim, Mühlheim, Deutschland; 5 Gemeinschaftspraxis Allgemeinmedizin, Hamburg, Deutschland; 6 Hautklinik, Universitätsklinikum Erlangen, Erlangen, Deutschland

**Keywords:** Debridement, Wundspülung, Schmerzen, Wundbehandlung, Wundheilung, Debridement, Wound irrigation, Pain, Wound therapy, Wound healing

## Abstract

Die heute in der Wundbehandlung verwendete Nomenklatur ist interdisziplinär und interprofessionell sehr unterschiedlich. Daher ist es ein Anliegen der Fachgesellschaft Initiative Chronische Wunde (ICW) e. V. bislang unklare Begriffe eindeutig und nachvollziehbar zu beschreiben. Von den Experten der ICW wurde daher in einem Konsensusverfahren als Débridement chronischer Wunden die Entfernung von anhaftendem, abgestorbenem Gewebe, Krusten oder Fremdkörpern aus Wunden bezeichnet. Hierfür gibt es verschiedene Therapieoptionen, die als autolytisches, biochirurgisches, mechanisches, osmotisches, proteolytisches/enzymatisches und technisches Débridement unterschieden werden können. Bei dem chirurgischen Débridement wird zudem zwischen meist ambulant durchführbaren scharfen Débridements wie beispielsweise kleineren chirurgischen Eingriffen und chirurgischen Débridements mit adäquater Anästhesie in einem Operationssaal differenziert. Als Wundspülung wird von der ICW die Entfernung von nicht haftenden Bestandteilen auf Wunden mit sterilen Lösungen bezeichnet.

Débridement und/oder Wundspülung sind oft der erste Schritt einer phasengerechten modernen Wundbehandlung. Mehrere Methoden eignen sich für die Anwendung einer kombinierten oder sukzessiven Therapie. Bei der Entscheidung, welche therapeutische Option hierbei zum Einsatz kommt, sollte eine Vielzahl individuell unterschiedlicher Faktoren in Abhängigkeit von den zu behandelnden Patienten, aber auch von den Therapeuten berücksichtigt werden. Die letztendliche individuelle Entscheidung für eine Methode sollte jeweils gemeinsam mit den Patienten getroffen und anschließend adäquat dokumentiert werden.

## Abstract (en)

The nomenclature used today in wound treatment varies widely across different disciplines and professions. Therefore, it is a mission of the professional association Initiative Chronische Wunde (ICW) e. V. to exactly and comprehensibly describe terms that were previously unclear. Therefore, the experts of the ICW defined in a consensus procedure debridement of chronic wounds as the removal of adherent, dead tissue, scabs or foreign bodies from wounds. There are various therapy options for this, which can be differentiated into autolytic, biosurgical, mechanical, osmotic, proteolytic/enzymatic and technical debridement. In the case of surgical debridement, a distinction is also made between sharp debridements that can usually be performed on an outpatient basis, such as minor surgical procedures, and surgical debridements with adequate anaesthesia in an operating theatre. Wound irrigation is defined by the ICW as the removal of non-adherent components on wounds with sterile solutions. Debridement and/or wound irrigation are often the first step in phase-appropriate modern wound treatment. Several methods are suitable for use in a combined or successive therapy. When deciding which therapeutic option to use, a number of individually different factors should be taken into account, depending on the patients to be treated but also on the therapists. The final individual decision for a method should be made together with the patient in each case and then adequately documented.

In die Behandlungsprozesse von Patienten mit chronischen Wunden sind Menschen vieler Berufsgruppen mit sehr unterschiedlichen Kompetenzen, Befugnissen und Weiterbildungen involviert. Als chronisch werden in diesem Kontext Wunden bezeichnet, die nach acht Wochen trotz Therapie nicht abgeheilt sind. Unabhängig von dieser zeitlich orientierten Definition, gibt es Wunden, die von Beginn an als chronisch anzusehen sind, da ihre Behandlung eine Therapie der weiterhin bestehenden Ursache erfordert. Hierzu gehören beispielsweise das diabetische Fußulkus (DFU), Wunden bei pAVK, Ulcus cruris venosum oder Dekubitus [10].

Derzeit gibt es zahlreiche verschiedene Definitionen, Stellungnahmen und Expertenartikel zu den Thematiken des Débridements chronischer Wunden [8]. Ohne allgemein akzeptierte Definitionen kommt es immer wieder zu Unsicherheiten hinsichtlich der zu verwendenden Nomenklatur. Diese Definitionen sind u. a. für eine einheitliche Dokumentation und Kommunikation aber auch für klinische Studien sehr wichtig. Es ist ein Anliegen der Fachgesellschaft Initiative Chronische Wunde (ICW) e. V., bislang unklare Begriffe eindeutig und nachvollziehbar zu beschreiben. Daher hat der Vorstand der ICW Vorschläge für Definitionen erstellt und diese mit dem interdisziplinär und interprofessionell besetzten wissenschaftlichen Beirat in einem Konsensusverfahren abgestimmt und überarbeitet.

Bei der kritischen Durchsicht der vorhandenen Literatur waren es in erster Linie die Empfehlungen der europäischen Wundheilungsorganisation European Wound Management Association (EWMA) von Strohal et al. aus dem Jahr 2013, die ein sehr gutes Grundgerüst für das aktuelle Positionspapier darstellen [28]. Allerdings gab es auch in den Empfehlungen der EWMA einige zentral wichtige Punkte, die aus unserer Sicht modifiziert und teilweise aktualisiert werden sollten. Darüber hinaus wurden auch die Inhalte des Expertenstandards des Deutschen Netzwerks für Qualitätsentwicklung in der Pflege (DNQP) sowie die aktuelle, seit 2017 abgelaufene Arbeitsgemeinschaft der Wissenschaftlichen Medizinischen Fachesellschaften (AWMF)-S3-Leitlinie Lokaltherapie chronischer Wunden bei Patienten mit den Risiken periphere arterielle Verschlusskrankheit, Diabetes mellitus, chronisch venöse Insuffizienz als zentral wichtige Beiträge kritisch diskutiert [7]. Da sich das praktische Vorgehen bei verschiedenen Arten von Wunden, wie beispielsweise postoperativen Wunden oder Verbrennungen, unterscheidet, wird im Folgenden der Fokus auf die relevanten Aspekte für die Behandlung von Patienten mit chronischen Wunden gelegt.

Es war somit die Intention der Experten der ICW, zu der Thematik des Débridements chronischer Wunden ein Positionspapier mit eindeutig beschriebener Nomenklatur zu erstellen, um eine nachvollziehbare Empfehlung als Grundlage beispielsweise für Behandlungen, Schulungen und Dokumentationen zu haben.

## Débridement

Als Débridement chronischer Wunden wird von der ICW die Entfernung von anhaftendem, abgestorbenem Gewebe, Krusten oder Fremdkörpern aus Wunden bezeichnet.

Als Erstbeschreiber des chirurgischen Débridements gilt der deutsche Chirurg Paul Leopold Friedrich, der 1898 als Débridement die Entfernung von Nekrose, Fibrin und Serokrusten sowie die Eröffnung von Wundtaschen beschrieb. Der Begriff Débridement stammt aus dem Französischen. Hier bedeutet „débrider“ wörtlich „abzäumen“ und meint die Entfernung von Überflüssigem. In diesem Kontext kann die Entfernung von Nekrosen, Detritus, Fibrin oder Fremdkörpern wie beispielsweise Resten von Wundverbänden genannt werden. Als Nekrose wird abgestorbenes, zuvor vitales Gewebe bezeichnet [10]. Im Expertenstandard des DNQP wird als Débridement die Entfernung von abgestorbenem Gewebe bezeichnet. Zentral wichtige Ziele des Débridements, die in der Literatur erwähnt werden, sind somit die Vermeidung und Bekämpfung von Wundinfektionen, Verbesserung der Beurteilbarkeit der Wunde sowie die Förderung der Wundheilung [7].

Das Débridement bezieht sich aus Sicht der ICW primär ausschließlich auf das Areal der Wundfläche. Allerdings kann es notwendig sein, dass ein chirurgisches Débridement auch den Wundrand mit einbezieht. Als Wundrand wird die Grenze zwischen Wunde und intaktem Epithel bezeichnet [10]. Andere therapeutische Maßnahmen am Wundrand oder der Wundumgebung können beispielsweise als Wundrand- oder Hautreinigung bezeichnet werden, sind aber kein direkter Bestandteil des Débridements. Auch die Abtragung von Hyperkeratosen auf ansonsten intakter Haut ist beispielsweise bei Patienten mit neuropathischen Wunden eine wichtige therapeutische Maßnahme, aber ebenfalls kein direkter Bestandteil des Débridements chronischer Wunden, da sich diese nicht innerhalb der Wundfläche befinden.

Für die praktische Durchführung des Débridements eignen sich bei Patienten mit chronischen Wunden grundsätzlich verschiedene therapeutische Methoden (Tab. 1).

**Table 1:** 

*Autolytisches Débridement z.* *B. mit Hydrogelen, Hydrofasern*
*Biochirurgisches Débridement mit Therapielarven*
*Chirurgisches Débridement*
– Scharfes Débridement, z. B. kleinere chirurgische Eingriffe
– Chirurgisches Débridement in (Voll‑)Narkose
*Mechanisches Débridement*
– Baumwollgazen
– Bürsten
– Faserpads, -tücher
– (Chirurgische) Pinzetten
– Raue/offen- und grobporige Schäume/Schwämme
*Osmotisches Débridement*
– Cadexomer-Granulate
– Honigpräparationen
– Zuckerderivate (Dextranomer-Pasten)
*Proteolytisches/enzymatisches Débridement, z.* *B. mit Kollagenase*
*Technisches Débridement*
– Hydrochirurgie, -lavage
– Laser
– (Leistungs‑)Ultraschall

### Scharfes und chirurgisches Débridement

Ein *chirurgisches Débridement* im engeren Sinne bezeichnet die vollständige Abtragung von avitalem Gewebe bis in intakte Gewebestrukturen. Hierbei kommt es meist zu Gewebe- und Gefäßverletzungen mit Blutungen. Eine Blutstillung kann beispielsweise durch manuelle Kompression oder die Verwendung hämostyptischer Wundauflagen wie beispielsweise Alginat- oder Chitosan-Verbände erfolgen [5]. Eine kurzfristige klinische Überprüfung der Blutungskontrolle wird empfohlen. Bei ausgeprägten Befunden sollte das chirurgische Débridement mit adäquater Anästhesie in einem Operationssaal erfolgen.

Als Wundrevision (nach Friedrich) wurde ursprünglich die vollständige chirurgische Exzision der Wunde einschließlich Wundgrund und Wundrand bezeichnet. Somit wird hier eine Variante des radikalen chirurgischen Débridements beschrieben, die heute auch als solche bezeichnet werden sollte.

Als *scharfes Débridement *werden in diesem Positionspapier zur besseren Abgrenzung von einem chirurgischen Débridement die Interventionen bis an den Rand des avitalen Gewebes in Wunden bezeichnet. Es erfolgt somit keine 100 %ige Abtragung des avitalen Gewebes. Hierbei kommt es meist nicht zu Blutungen. Dieses Vorgehen ist grundsätzlich auch ambulant durchführbar. Oft ist im Anschluss an ein scharfes Débridement noch eine Behandlung mit anderen Débridementmethoden sinnvoll.

Für ein scharfes bzw. chirurgisches Débridement werden meist Skalpell, Pinzette und/oder Präparierscheren verwendet. Die scharfe Umschneidung sollte grundsätzlich mit einem Skalpell erfolgen. Für die Präparation können Scheren genutzt werden. Ringkürretten sind den scharfen Löffeln vorzuziehen. Das scharfe bzw. chirurgische Débridement ist je nach Schmerzsituation des Patienten, Verfügbarkeit und Qualifikation der Mitarbeiter meist die Methode der ersten Wahl, um möglichst rasch auch größere chronische Wunden zu débridieren.

### Mechanisches Débridement

Als mechanisches Débridement werden verschiedene Therapieformen zusammengefasst, bei denen locker haftende Wundbestandteile wie beispielsweise Fibrin weitestgehend atraumatisch aus den Wunden entfernt werden. Sterile Baumwollkompressen sind hierfür eine traditionell verwendete, sichere Methode [21]. Darüber hinaus gibt es (chirurgische) Pinzetten, (Micro‑)Faserpads und -tücher sowie raue/offen- und grobporige Schäume/Schwämme, die für ein mechanisches Débridement genutzt werden können [2, 23]. Eine Modifikation ist die sog. Nass-Trocken-Phase(„wet-to-dry“)-Methode, bei der angefeuchtete Baumwollkompressen auf die Wunde gelegt werden. Das avitale Gewebe trocknet aus, verhärtet und verklebt mit der Kompresse, die dann mit dem anhaftenden Material entfernt wird [27].

Das mechanische Débridement kann in der Regel sicher und mit geringem Zeitaufwand durchgeführt werden. Lediglich bei der Wet-to-dry-Methode sind längere Zeitintervalle für die Durchführung notwendig. Im deutschsprachigen Raum finden sich meist Empfehlungen von 15–20 min für die Durchführung der Nassphase [17].

Synonym werden für ein mechanisches Débridement oft auch die Begriffe Wundreinigung oder Wundsäuberung verwendet. Um eine eindeutige Zuordnung zu ermöglichen, empfiehlt die ICW, für diese Prozeduren ausschließlich den Begriff mechanisches Débridement zu verwenden.

### Biochirurgisches Débridement

Als Biochirurgie wird die Behandlung von Wunden mit steril gezüchteten Therapielarven, meist aus der Gattung *Lucilia sericata*, bezeichnet. Die Larvenprodukte sind gemäß Arzneimittelgesetz (AMG) zugelassene, verschreibungspflichtige Fertigarzneimittel. Die Larven werden im Rahmen des biochirurgischen Débridements auf Wunden entweder als sog. Freiläufer oder als Biobag eingesetzt. Durch die extrakorporale Verdauung kommt es zu einer sehr selektiven Lyse von nekrotischem Gewebe und Bakterien [14]. Hierdurch kann es in Einzelfällen zu verzögert auftretenden Arrosionsblutungen unter dem Verband kommen. Daher ist eine regelmäßige klinische Kontrolle erforderlich.

Der Begriff biochirurgisches Débridement ist den oft synonym verwendeten Begriffen wie (Fliegen‑)Larven- oder Madentherapie vorzuziehen, da dieser Terminus bei den meisten Patienten und Angehörigen nicht negativ bzw. abschreckend besetzt ist.

### Autolytisches Débridement

Bei dem autolytischen Débridement werden u. a. körpereigene proteolytische Enzyme freigesetzt und Phagozyten aktiviert. Wundprodukte für das autolytische Débridement sind beispielsweise Hydrogele, Gelkompressen, Alginate, Hydrofasern oder Hydrokolloide. Ein autolytisches Débridement ist selektiv, schmerzarm, einfach und sicher durchzuführen. Es werden allerdings längere Zeitspannen im Vergleich zu physikalischen Methoden benötigt.

### Proteolytisches/enzymatisches Débridement

Für ein proteolytisches Débridement werden meist Enzyme eingesetzt, die Peptidbindungen hydrolysieren. Wörtlich wird als Proteolyse der Abbau von Eiweiß bezeichnet. Für die Wundbehandlung werden beispielsweise Produkte mit Bromelain, Kollagenase oder Streptokinase und Streptodornase genutzt [22, 26]. Auch das proteolytische Débridement ist eher schmerzlos, einfach und sicher durchzuführen. Allerdings sind meist tägliche Verbandwechsel erforderlich.

### Osmotisches Débridement

Bei einem osmotischen Débridement werden absorbierende Wundprodukte eingesetzt. Anwendung finden hier meist hyperosmolare Produkte wie beispielsweise Zuckerderivate (Dextranomer-Pasten), Honigpräparationen oder Cadexomer Granulate [30]. Es ist unbedingt zu beachten, dass es sich bei den eingesetzten Produkten für ein osmotisches Débridement um dafür zugelassene Wundprodukte und nicht beispielsweise um unsterile Lebensmittel handelt. Ein osmotisches Débridement kann einfach und sicher durchgeführt werden. Die wichtigste Nebenwirkung sind teils ausgeprägte Schmerzen, die bereits während der Therapie und bei der oft aufwendigeren Entfernung der Verbände auftreten können.

### Technisches Débridement

Für ein sog. technisches Débridement werden beispielsweise Hydrochirurgie, -Lavage, Laser oder (Leistungs‑)Ultraschallgeräte eingesetzt [8]. Unterstützend kann auch eine Vakuumtherapie genutzt werden [1]. Da einige der technischen Débridementmethoden auch bis in gesundes Gewebe schneiden können, entsprechen sie dann einem chirurgischen Débridement [16]. Nachteile dieser innovativen technischen Débridementoptionen sind u. a. die regelmäßig notwendige Desinfektion der Geräte und teilweise auch der durch die Behandlung kontaminierten Umgebung, Zeitaufwand, Schmerzen und die oft sehr hohen Kosten.

### Weitere Débridementoptionen

Es gab in der Vergangenheit weitere, meist zytotoxische Methoden wie beispielsweise die chemischen Débridements mit Säuren [19], bei denen insbesondere bei Verbrennungswunden unselektiv Gewebe zerstört wurde. Diese Methoden sind heute in der Behandlung von Patienten mit chronischen Wunden als obsolet zu betrachten und somit nicht mehr anzuwenden.

## Analgesie

Vor einem Débridement sollten auch immer Überlegungen zu einer individuellen, adäquateren Analgesie erfolgen. Schmerzen sollten dafür regelmäßig mittels geeigneter Messinstrumente wie beispielsweise der visuellen Analogskala (VAS) oder numerischen Rangskala (NRS) objektiviert werden. Für kleinere Eingriffe reicht oft schon die topische Applikation beispielsweise einer lokal anästhesierenden Creme mit Lidocain und Prilocain [4]. Hierbei ist auf eine adäquate Mindesteinwirkzeit zu achten, die nicht unter 30–60 min liegen sollte. Insbesondere bei chirurgischen Débridements sollte mit den Patienten auch die Option der Durchführung in Vollnarkose besprochen werden.

## Komplikationen

Komplikationen können sich insbesondere bei chirurgischen Débridements durch ausgeprägte Blutungen ergeben. Daher sind Gerinnungsstörungen und Therapien mit Antikoagulanzien zu erfragen und bei der Planung der Interventionen zu berücksichtigen.

Zudem sollte beachtet werden, dass es Krankheitsbilder gibt, die sich durch ein Débridement verschlechtern können. Dieses sog. Pathergiephänomen findet sich beispielsweise in der inflammatorischen Phase des Pyoderma gangraenosum [20] Wenn die Inflammation medikamentös kontrolliert ist, dann kann und sollte auch bei solchen Wunden ein Débridement durchgeführt werden [3].

## Wundspülung

Als Wundspülung wird von der ICW die Entfernung von nicht haftenden Bestandteilen mit sterilen Lösungen bezeichnet. Neben Resten von Verbandmitteln sind es oft (angetrocknete) Wundexsudate, die hierbei entfernt werden [24]. Als Wundexsudat werden alle Flüssigkeiten bezeichnet, die von einer Wunde freigesetzt werden. In Abhängigkeit des Wundzustandes kann diese Lymphe, Blut, Proteine, Keime, Zellen und Zellreste beinhalten [10]. Im Expertenstandard „Pflege von Menschen mit chronischen Wunden“ wird als Wundreinigung die Entfernung von avitalem Gewebe, überschüssigem Exsudat und metabolischen Abfallstoffen bezeichnet [7]. Eine Wundspülung kann grundsätzlich auch das Ausduschen von Wunden sein, wenn endständige Wasserfilter genutzt werden [25]. Eine weitere Alternative ist die Vakuumtherapie mit Instillation [15]. Bei der Verwendung von Leitungswasser sind unbedingt die aktuellen Hygienerichtlinien des Robert Koch-Instituts (RKI) zu beachten. Wenn Antiseptika für die Wundspülung verwendet werden, dann ausschließlich zur Reduktion von Keimen bei Vorliegen einer bestehenden oder drohenden Wundinfektion [18]. Weitere hygienische Aspekte werden in der aktuellen Hygieneleitlinie der ICW ausführlicher dargestellt.

Um eine klare Abgrenzung zum Débridement zu gewährleisten, empfiehlt die ICW, ausschließlich den Begriff Wundspülung zu verwenden.

## Kodierung

Der Operationen- und Prozedurenschlüssel (OPS) ist in Deutschland die offizielle Klassifikation zum Verschlüsseln von Operationen, Prozeduren und medizinischen Maßnahmen. Diese Klassifikation wird durch das Deutsche Institut für Medizinische Dokumentation und Information (DIMDI) im Auftrag des Bundesministeriums für Gesundheit erstellt und aktualisiert. Hierbei handelt es sich um die Adaptation der Internationalen Klassifikation der Prozeduren in der Medizin (ICPM) der Weltgesundheitsorganisation (WHO). Diese OPS-Ziffern sind eine wichtige Grundlage für die Leistungsabrechnung sowohl für die stationären als auch die ambulant erbrachten ärztlichen Leistungen.

Unter der OPS-Ziffer 5–896 wird das chirurgische Wunddébridement beschrieben, was hier synonym auch als „Wundtoilette“ bezeichnet wird. Die ICW empfiehlt in der eigenen Dokumentation, hierbei ausschließlich den Begriff chirurgisches Wunddébridement zu verwenden und den Terminus „Wundtoilette“ aufgrund einer ggf. negativen Besetzung des Wortes bei den Betroffenen und Angehörigen nicht mehr zu verwenden. In dem aktuellen OPS-Code wird ein Débridement als „kleinflächig“ bezeichnet, wenn die Länge der behandelten Wunde maximal 3 cm oder die Fläche maximal 4 cm^2^ beträgt. Wenn die mittels Débridement behandelten Flächen größer sind, wird die Maßnahme entsprechend als „großflächiges Débridement“ dokumentiert. In der inhaltlichen Beschreibung heißt es „Entfernung von erkranktem Gewebe an Haut und Unterhaut“. Es wird darauf hingewiesen, dass die Entfernung von erkranktem Gewebe an Haut und Unterhaut ohne Anästhesie im Rahmen eines Verbandwechsels nicht mit dieser OPS-Ziffer kodiert werden kann. Die Kodierung des chirurgischen Wunddébridements setzt somit im OPS-Code explizit eine Allgemein- oder Regionalanästhesie oder eine lokale Infiltrationsanästhesie voraus. Als Ausnahme wird hier aber eine neurologisch bedingte Analgesie bezeichnet, so wie diese beispielsweise bei Patienten mit neuropathischen Wunden bei DFU vorliegen können. Diese Beschreibungen entsprechen somit auch den aktuellen Empfehlungen der Nomenklatur der ICW.

Die durch die ambulante Pflege durchgeführten therapeutischen Maßnahmen im Rahmen einer Wundversorgung können bei der Notwendigkeit eines Débridements und/oder einer Wundspülung nicht zusätzlich abgerechnet bzw. vergütet werden. In der aktuellen Häuslichen Krankenpflege(HKP)-Richtlinie ist die Leistung, „Desinfektion und Reinigung, Spülen von Wundfisteln“ unter Nummer 31 Wundversorgung bei akuten Wunden und Nummer 31a Wundversorgung einer chronischen und schwer heilenden Wunde mit zu erbringen [11].

## Diskussion

Eingebunden in Überlegungen zu der Behandlung der zugrunde liegenden Ursachen, ist das Débridement meist der erste Schritt in einer phasengerechten Therapie chronischer Wunden. Entsprechend dem MOIST-Konzept [9] der Wundbehandlung werden Débridement und Wundspülung im Kontext des Gewebemanagements (T = „tissue management“) bzw. der Wundgrundkonditionierung beschrieben (Tab. 2). Bei vielen der genannten Verbandmittel sollte zusätzlich auf einen Schutz der Wundumgebung geachtet werden, da sonst Mazerationen auftreten können [6].

**Table 2:** 

M	„Moisture balance“ = Exsudatmanagement
O	„Oxygen balance“ = Sauerstoffzufuhr
I	„Infection control“ = Infektionskontrolle
S	„Support“ = Unterstützung des Heilungsprozesses
T	„Tissue management“ = Gewebemanagement

Die sehr wichtige Bedeutung des Débridements als Bestandteil der Wundbehandlung wurde mittlerweile in zahlreichen wissenschaftlichen Studien gut belegt. Allerdings sollten insbesondere chirurgische Débridements nicht dauerhaft und ohne Selektion durchgeführt werden, da es sonst zu Fibrosierung bzw. Sklerosierung des Wundgrundes und somit zu einer Verschlechterung der Wundheilung kommen kann. Die Cochrane-Gruppen untersuchten in ihren Metaanalysen die klinischen Studien zum Wunddébridement bei verschiedenen chronischen Wunden. Für das Ulcus cruris venosum wurden 2015 insgesamt zehn RCTs [13] und für das DFU sechs RCTs [12] gefunden. Hier fanden sich einige wissenschaftliche Hinweise darauf, dass die Durchführung eines Débridements die Wundheilung bei diesen chronischen Wunden unterstützen kann.

In einer retrospektiv analysierten Studie mit 154.644 Patienten und 364.534 Wunden verschiedener Entitäten zeigte sich, dass je häufiger ein Débridement durchgeführt wurde, desto schneller heilten die Wunden [29]. Hier gab es Patienten, bei denen täglich Débridements durchgeführt wurden. Diese addierten sich zu einer Gesamtzahl von bis zu 138 dokumentierten Débridements. Die Reinigung der Wunden u. a. mit sterilen Baumwollkompressen wurde in dieser Studie, so wie auch von der ICW empfohlen, als mechanisches Débridement dokumentiert. Diese viel beachtete Studie ist ein gutes Beispiel dafür, dass eindeutig geklärte Begriffe auch die Grundlage für die bessere Vergleichbarkeit von wissenschaftlichen Studien sind.

Das einzig richtige Débridement chronischer Wunden gibt es nicht. So individuell die Patienten und die Ursachen der zugrunde liegenden Wundheilungsstörungen sind, so unterschiedlich sinnvoll kann auch der Einsatz der verschiedenen Methoden des Débridements sein. Bei der individuell zu treffenden Auswahl für ein geeignet erscheinendes Débridement sollten gemeinsam mit den Patienten verschiedene Faktoren berücksichtigt werden (Tab. 3). In Einzelfällen kann es sinnvoll sein, verschiedene Methoden des Débridements sukzessive zu nutzen.

**Table 3:** 

Behandlungsumfeld
Kofaktoren und Komorbiditäten der Patienten
Kompetenzen der Therapeuten
Präferenzen der Patienten und Therapeuten
Rechtliche Aspekte und Vorschriften, ggf. Leitlinien
Schmerzen der Patienten
Verfügbarkeit und Ressourcen
Wirtschaftliche Aspekte

## Fazit für die Praxis

Débridement und/oder Wundspülung sind oft der erste Schritt einer phasengerechten modernen Wundbehandlung. Welche therapeutische Option hierbei zum Einsatz kommt, ist von einer Vielzahl individuell unterschiedlicher Faktoren abhängig. Mehrere Methoden eignen sich auch für Anwendung einer kombinierten oder sukzessiven Therapie. Die letztendliche individuelle Entscheidung über die durchzuführende Therapie sollte jeweils mit den Patienten gemeinsam getroffen werden.
